# An automated and simple method for brain MR image extraction

**DOI:** 10.1186/1475-925X-10-81

**Published:** 2011-09-13

**Authors:** Haiyan Zhang, Jiafeng Liu, Zixin Zhu, Haiyun Li

**Affiliations:** 1 College of Biomedical engineering, Capital Medical University, Beijing 100069, P.R.China; 2 Department of Radiology, Xuanwu Hospital Capital Medical University, Beijing 100053, P.R.China

## Abstract

**Background:**

The extraction of brain tissue from magnetic resonance head images, is an important image processing step for the analyses of neuroimage data. The authors have developed an automated and simple brain extraction method using an improved geometric active contour model.

**Methods:**

The method uses an improved geometric active contour model which can not only solve the boundary leakage problem but also is less sensitive to intensity inhomogeneity. The method defines the initial function as a binary level set function to improve computational efficiency. The method is applied to both our data and Internet brain MR data provided by the Internet Brain Segmentation Repository.

**Results:**

The results obtained from our method are compared with manual segmentation results using multiple indices. In addition, the method is compared to two popular methods, Brain extraction tool and Model-based Level Set.

**Conclusions:**

The proposed method can provide automated and accurate brain extraction result with high efficiency.

## Background

Brain extraction which segments magnetic resonance (MR) head images into brain and non-brain region is often required for analyses of neuroimage data. Accurate and automated brain extraction plays an important role in the analyses because brain region should be isolated before other processing algorithms such as tissue classification, registration or cortical surface reconstruction can be made [1-3]. For example, functional images such as Functional magnetic resonance image (FMRI) and Positron Emission Tomography (PET) image usually contain few non-brain tissues, whereas high resolution MR images often contain some non-brain tissues(i.e., skin, fat, muscle, etc.), and if the non-brain tissues of MR images can be accurately removed beforehand, the registration robustness will be improved greatly [2]. Furthermore, as a pre-processing step, brain extraction is usually performed before a full segmentation of the brain region into grey matter (GM), white matter (WM), and cerebrospinal fluid (CSF), so that the segmentation problem can be simplified [4,5]. On the other hand, brain extraction is also a difficult and time-consuming pre-processing step performed in neuroimage analysis due to the complexity of human brain anatomy and weak boundaries between brain and non-brain tissues.

Ma et al. [6] pointed out that researchers should combine the application background with practical requirements to design a proper algorithm for a medical image segmentation task. Although many brain extraction algorithms (BEAs) have been proposed to accurately segment brain from non-brain tissues, their segmentation quality varies greatly and has important influence on the results of subsequent image analysis. Boesen et al. [7] compared the performance of four BEAs and concluded that the brain extraction tool (BET) and the brain surface extractor (BSE) was significantly faster than the statistical parametric mapping(SPM) or Minneapolis consensus strip(McStrip). Compared to two different manual strip-masks, however, McStrip outperformed BET, SPM and BSE based on the Correct Boundary and Pertinent Boundary criteria and misclassified the least number of brain voxels. These popular methods have both their advantages and weaknesses, and none of them can be accurate and robust enough for large-scale neuroimage analysis [7,8]. Subsequent research work are aimed at developing fully automated, accurate and robust BEAs for MR images. To facilitate large-scale neuroimage analysis, Zhuang et al. [9] developed a new automatic BEA called the model based level set method (MLS) which can provide robust performance for large-scale neuroimage analysis. As the number of subjects increases and the real-time image processing for clinical application develops, the need for fully automated, simple and fast brain extraction algorithms will become critical. In this paper, we first proposed a new method satisfying the requirement of both fully automated brain extraction and accurate brain extraction result; then we summarized the experimental results, evaluation, and comparison of our method to BET and MLS; finally, we discussed the advantages and disadvantages of our method for brain MR image extraction.

## Methods

We developed an accurate and simple brain extraction method using an improved geometric active contour model (GAC) which can not only solve the boundary leakage problem but also is less sensitive to intensity inhomogeneity. The proposed brain extraction method comprises three major steps: image intensity parameters are first estimated and a binary image of the head is calculated for the following segmentation procedures. Then the initial contour is automatically determined within the brain region. Finally, the proposed geometric active contour model is applied to extract the brain region on each of the slices.

### The proposed GAC model with a new local region-based signed pressure force function for brain MR image extraction

Geometric active contour models [10,11] are based on the theory of curve evolution and the level set method [12]. Let *ϕ(x, t) *be a 3-D scalar function whose zero level set defines the geometric active contour. The traditional geometric active contour formulation is written below [10,11]:

(1) $$\frac{\partial \phi }{\partial t}=c\left(k+v_{0}\right)\left|\nabla \phi \right|$$

where *k *is the curvature and *v_0 _*is a constant, and

(2) $$c=c\left(x\right)=\frac{1}{1+\left|\nabla G_{\sigma }*I\left(x\right)\right|}$$

is an edge potential function derived from the image. In Eq.(1), the product *c(k+v_0_) *determines the overall evolution speed of level sets *ϕ *along their normal direction. The curvature *k *has the effect of smoothing the contour, while *v_0 _*has the effect of shrinking or expanding contour at a constant speed. The speed of contour evolution is coupled with the image data through a multiplicative stopping term *c*. This scheme works well for objects that have good contrast, but when it is used for detecting boundaries between brain and non/brain tissues, this contour tends to leak through the boundary or fail to reach the desired boundary. To solve the so-called boundary leaking problem, Zhang et al. [13] proposed a region-based active contour model with a region-based signed pressure force (SPF) function which can efficiently stop the contours at weak or blurred edges. The SPF function is also called the region function which is derived from the image and has values in the range [-1, 1]. The region function modulates the sign of the pressure forces using region information so that the contour shrinks when it is outside the object of interest or expands when it is inside the object. The model constructed by Zhang et al. [13] only used the image statistical information of the entire region inside and outside the contour, which can't successfully segment brain MR images with intensity inhomogeneity. However, intensity inhomogeneity often occurs in MR images due to technical limitations or motion artifacts and may cause difficulties in image segmentation. Li et al. [14] proposed a region-based local binary fitting (LBF) model which utilized the local intensity information in local regions to solve the problem caused by intensity inhomogeneity. To solve the boundary leakage and intensity inhomogeneity which were both common in brain MR image extraction, we constructed a new local region-based SPF function which utilized two smooth constants that locally approximated the image intensities inside and outside the contour as follows:

(3) $$spf\left(x\right)=\frac{G_{\sigma }\left(x\right)*I\left(x\right)-\frac{f_{1}+f_{2}}{2}}{\max\left(\left|G_{\sigma }\left(x\right)*I\left(x\right)-\frac{f_{1}+f_{2}}{2}\right|\right)},x\in \Omega$$

where *G_σ _*is the Gaussian kernel with standard deviation *σ *and * denotes the convolution operator. In Eq.(3), We take the maximum absolute value of the numerator as the denominator, which can ensure the Eq.(3) has values in the range [-1,1], so Eq.(3) can serve as a SPF function. *f_1 _*and *f_2 _*are two smooth constants defined as follows:

(4) $$\begin{array}{c} f_{1}=\frac{\int_{\Omega }G_{\sigma }*\left[H_{\varepsilon }\left(\phi \right)I\right]d\Omega }{\int_{\Omega }H_{\varepsilon }\left(\phi \right)d\Omega }\;,\\ f_{2}=\frac{\int_{\Omega }G_{\sigma }*I-G_{\sigma }*\left[H_{\varepsilon }\left(\phi \right)I\right]d\Omega }{\int_{\Omega }1-H_{\varepsilon }\left(\phi \right)d\Omega } \end{array}$$

where *H(ϕ) *is the Heaviside function which is generally approximated by a smooth function *H_ε _*defined as follows:

(5) $$H_{\varepsilon }\left(x\right)=\frac{1}{2}\left[1+\frac{2}{\pi }\arctan\left(\frac{x}{\varepsilon }\right)\right]$$

where *ε *is a positive constant. *f_1 _*and *f_2 _*of Eq.(4) can be regarded as the weighted averages of image intensities in a Gaussian window inside and outside the contour, respectively. Therefore, the proposed SPF function can not only utilize the locally statistical information inside and outside the contour to control the evolution but also use the smoothing effect of Gaussian filter, which is thus less sensitive to noise and intensity inhomogeneity. Substituting the SPF function in Eq.(3) for the edge potential function *c(x) *in Eq.(1), the level set formulation of the proposed model for brain extraction is as follows:

(6) $$\frac{\partial \phi }{\partial t}=spf\left(x\right)\left(k+v_{0}\right)\left|\nabla \phi \right|\;,\;x\in \Omega$$

The proposed geometric active contour model utilizes the new local region-based SPF function to solve the boundary leaking problem and intensity inhomogeneity which traditional geometric active contour models fail to solve. So our model not only works well for objects that have good contrast but also for objects with weak boundary such as brain surface.

### Estimation of image intensity parameters and binary image of the head

**Figure 1:**
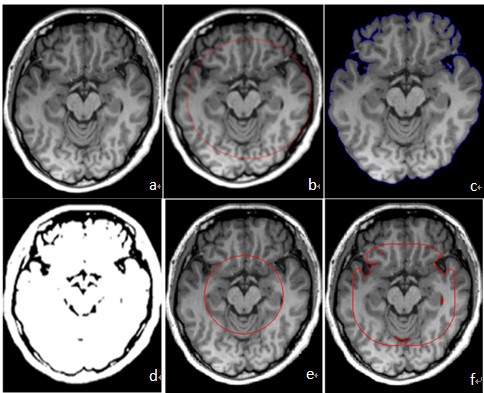
**Results comparison of different initial contour**. Figure. 1. (a) The original MR image. (b)an appropriate initial contour close enough to the brain surface. (c) satisfactory segmentation result only after 20 iterations with the initial contour of (b). (d) the binary image of the head with threshold t which can roughly separate brain tissue from non-brain tissue. (e)an inappropriate initial contour far away from the brain surface. (f) unsatisfactory segmentation result only after 20 iterations with the initial contour of (e).

We estimate the effective intensity range in the same way as the work of Smith [2]. An effective intensity range [*t_1_, t_2_*] is determined to ignore the voxels with unusual intensities, where *t_1_, t_2 _*are the intensity values in the histogram chosen such that the accumulated number of voxels reaches 2% and 98%, respectively. Subsequently, a threshold *t *is chosen empirically to separate the background and the skin or muscle tissue that covers the head which can be calculated as:

(7) $$t=\left(t_{2}-t_{1}\right)\;T_{c}+t_{1}$$

where *T*c is a constant different for axial, coronal and sagittal orientation. The value of *T_c _*is determined according to image data empirically and sometimes several local thresholds may be needed to solve leakage problem. With the threshold *t*, we can get the binary image of head as shown in Figure 1.(d), which is useful for the determination of initial contour.

### Automatic initialization

Automatic initialization is required for fully automated brain extraction and can affect the accuracy of the segmentation results. Some non-brain tissues have intensity characteristics similar to brain tissues and could produce errors in the segmentation if the initial curve includes them [8]. Smith [2] used the centre of the head volume (which contains both brain and non-brain regions) and the half radius of the head volume to initialize a sphere. Zhuang et al. [9] initialized the zero level curve at the centre of the brain, with a diameter just small enough so that the initialization methods was completely within the brain surface. However, a good initialization should set the initial active contour close enough to the final targeted surface to avoid local minima. Besides, too small diameter also affects computational efficiency, therefore we try to initialize the initial contour close enough to the true boundaries between brain and non-brain region. Liu et al. [15] initialized an ellipse centred at the brain centroid and the lengths of its axes set to be 0.7 times the length and width of the brain bounding box. Because the determination of brain centroid necessitates the calculation of the first order image moment of voxels in the required range, which increases the computational time, we proposed a new initial contour generating method to automatically set the initial contour not only in brain region but also close enough to the true boundaries. Figure 1.(b) and 1(c) show that the segmentation result is satisfactory after only 20 iterations if the appropriate initial contour is close enough to the brain surface; Figure 1.(e) and 1(f) show that the segmentation result is not satisfactory after 20 iterations with the inappropriate initial contour. If with the inappropriate initial contour as Figure 1.(e), the segmentation with good result would need 80 iterations. Figure 1 shows that our initial contour method can greatly reduce iteration times, which means that our initialization method is computationally efficient. Furthermore, in traditional level set methods, the level set function, *ϕ*, is initialized as a signed distance function *ϕ_0 _*for computational efficiency and re-initialization has been extensively used as a numerical remedy for maintaining stable curve evolution [12]. However, in practice, the re-initialization process is complicated and expensive. In this work, the initial function, *ϕ_0 _*is defined as a binary level set function as in the work of Lie [16]. Such initialization is very simple to implement in practice and can greatly improve computational efficiency. The initializing method is as follows:

1. Find the most left and right voxels automatically by searching all the voxels with the maximum intensity of the binary image of the head.

2. Compute the distance *d_l-r _*between the most left and right voxels.

3. Approximate the shape of the brain in axial as a square with its length set to be the distance *d_l-r_*, and the shape of the brain in coronal or sagittal orientation as a rectangle with a 8:7 and 4:3 ratio of width (equal to the distance *d_l-r _*) to height, respectively.

4. Initialize the zero level set curve as a circle and the initial circle is positioned at the center of the square or rectangle with its radius, r, equal to one third of the length of the square side(for axial orientation) or one third of the width of the rectangle(for coronal and sagittal orientation).

5. To simplify the initialization of the level set function, we use a binary level set function as in the work of Lie [16]. Each level set function can only take two values at convergence, then the initial function *ϕ_0 _*is defined as:

(8) $$\phi_{0}\left(x,\;y\right)=\left\{\begin{array}{c} -\rho \;\;\left(x,\;y\right)\in \text{int}\;\left(\Omega_{1}\right)\\ \rho \;\;\;\;\left(x,\;y\right)\in out\left(\Omega_{1}\right) \end{array}\right)\;$$

where *ρ *is a positive constant, *ϕ_0 _(x, y) *denotes *ϕ(x, y, t) *at t = 0, Ω is the enclosing interface. With such initialization of the level set function, not only the re-initialization procedure is completely eliminated, but also the level set function *ϕ *is no longer required to be initialized as a signed distance function.

**Figure 2:**
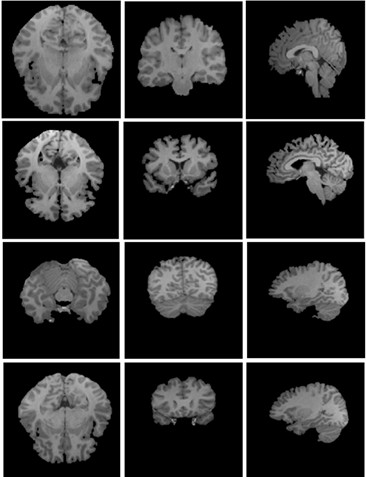
**Brain extraction results of four normal T1-weighted MR brain images**. Columns from left to right is shown in axial, coronal and sagittal orientations respectively.

We applied the proposed geometric active contour model to extract the brain regions in axial, coronal and sagittal orientations as shown in Figure 2. Because the brain region in some of the first or last slices is very small, to improve computational efficiency, the segmentation starts with approximately the slice at one tenth of the volume and ends with the slice at the nine tenths in each orientation. For example, if the volume consists 60 slices, our algorithm segment from the 6th slice and end with 54th slice. Furthermore, because the brain boundaries of neighbouring slices are usually similar and the evolved contour of current slice provides a good initial for the neighbouring ones, we use the brain extraction result from the current slice to initialize the contour in adjacent slices [17]. This can save computation time and improve the efficiency and accuracy of the results.

### Correction of leakage through weak boundaries based on local thresholds estimation

**Figure 3:**
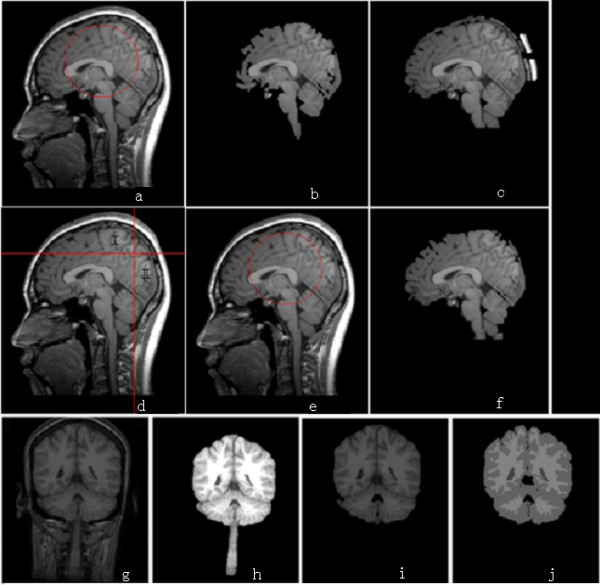
**Correction of weak boundary leakage**. (a) original MR image with proper initial contour. (b) over-segmentation results with single threshold. (c) leakage through weak boundaries with single threshold. (d) two parts with two different thresholds. (e) original MR image with an appropriate initial contour. (f) segmentation results with two local thresholds. (g) original MR image provided by IBSR. (h) segmentation results of MLS in which leakage occurs. (i) segmentation results of our method without leakage. (j) expert segmentation results provided by IBSR.

Weak boundaries between brain tissues and surrounding tissues are often seen in brain MR images, which result in leakage through these boundaries in brain extraction. For example, in areas where there is a lack of CSF, some non-brain tissues such as muscle tissues close to the cerebellum have intensities similar to brain tissues and could produce weak gradient between them, making it difficult to segment them. To correct the leakage of the evolving contour through weak boundaries, Zhuang et al. [9] detected the leakage through a weak boundary first by calculating the Jaccard coefficient, then increased the weight of the mean curvature force *Fcurv *to prevent high curvature, and lastly segmented the same slice again. However, the correction step is not completely effective and also time-consuming as shown in Figure 3.(h). In this work, we try to solve the problem with a higher efficiency. So we propose an method to correct effectively the leakage of weak boundaries based on local thresholds estimation. Our method to prevent such leakage comprises two major steps. First, we confirm if leakage occurs or not during segmenting process. By observing, we find such leakage through weak boundaries often occurs at some points with high curvature (shown in Figure 3.(c)), so we search such points and, if the number of these points reaches a pre-set value which is determined according to image data empirically, we estimate that segmentation leakage will occur, so the segmentation will be stopped immediately. Then, a new segmentation for the same slice will start again based on the local thresholds estimation. In local thresholds estimation, the brain region is divided into several parts with two different thresholds which is used to separate the background and other non-brain tissues as mentioned previously. As illustrated by Figure 3.(d), the thresholds of part I and part II are the same but higher than those of the other parts. The reason to make such improvement lies in the fact that, in some areas, the CSF is often thinner than other parts, leading to similar intensities of non-brain tissues with brain tissues and the leakage through weak boundaries is liable to occur. As a result, these parts are separated with a higher threshold, which can reveal more details of the weak boundary and lower the risk of the segmentation leakage. Experimental results show that local thresholds estimation can solve the boundary leaking problem effectively as shown in Figure 3.(f). Figure 3.(h), (i), and 3(j) also list the segmentation results with leakage of MLS, our segmentation results without leakage, and expert segmentation results, respectively.

## Results and Discussion

**Figure 4:**
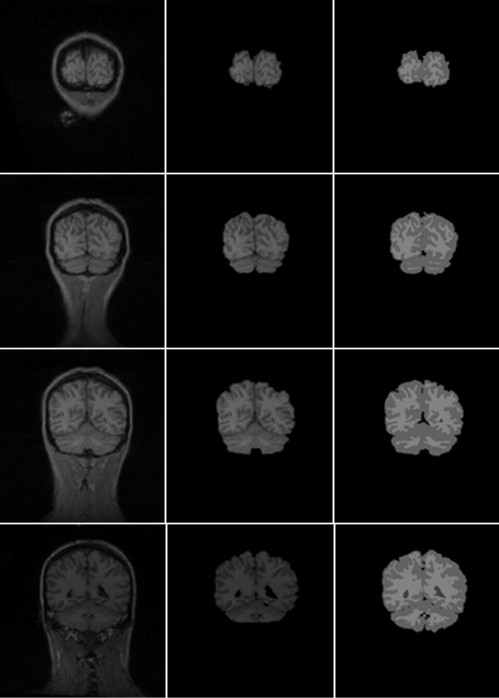
**Brain extraction results of four sample normal adult datasets downloaded from the IBSR shown in coronal orientation**. Columns from left to right: raw image, brain extraction results of our method and manual extraction provided by ISBR.

Our algorithm was implemented in Matlab 7.0 on a 2.1 GHz Intel Core 2 Duo T6500 PC with 2 G memory. We tested our algorithm on 10 normal T1 MR brain images obtained from Hospital with informed consent from all subjects. Each volume consisted of 176 slices, 448 × 512 pixels per slice. The slice resolution is 0.5 × 0.5 mm^2 ^and the slice thickness is 1 mm. We used the following default setting of the parameters: σ = 1.5, time step Δ*t *= 1, *ρ *= 1, *v_0 _*= 5. Figure 2 shows the final results on four sample images displayed in three orientations. To measure the extraction accuracy of our algorithm, 10 normal MRI brain data sets and the corresponding manual segmentations were obtained from the Internet Brain Segmentation Repository (IBSR) developed by the Centre for Morphometric Analysis (CMA) at Massachusetts General Hospital. Each volume has around 65 coronal slices, with 256 × 256 pixels per slice. The slice resolution is 1.02 × 1.04 mm^2^, and slice thickness is 3.1 mm. Figure 4 shows the final results of our method on eight sample volumes as well as manual segmentation results displayed in coronal orientation. We also computed the *sensitivity, specificity, Jaccard *index, *Dice *index and the *FP_Rate *of our segmentation results using the manual segmentation results provided by the IBSR (shown in Table 1).

**Table 1:** Performance comparison of BET, MLS and the proposed method for multiple indices using the IBSR data sets

Method	Sensitivity	Specificity	Jaccard	Dice	FP_rate
BET	***0.999(0.001)***	0.982(0.005)	0.896(0.045)	0.945(0.026)	0.115(0.063)
MLS	0.982(0.03)	0.991(0.008)	***0.925(0.041)***	***0.961(0.022)***	0.069(0.055)
Our method	0.973(0.01)	***0.993(0.003)***	0.923(0.022)	0.960(0.012)	***0.05(0.022) ***

mean(standard deviation) for multiple indices. The best performance for each index is in bold and italics

* FP_Rate is the number of voxels incorrectly classified as brain tissue by the automated algorithm divided by manually segmented brain masks. Therefore, if the other indices are same, then the lower the FP_Rate coefficient, the more accurate the segmentation results.

**Figure 5:**
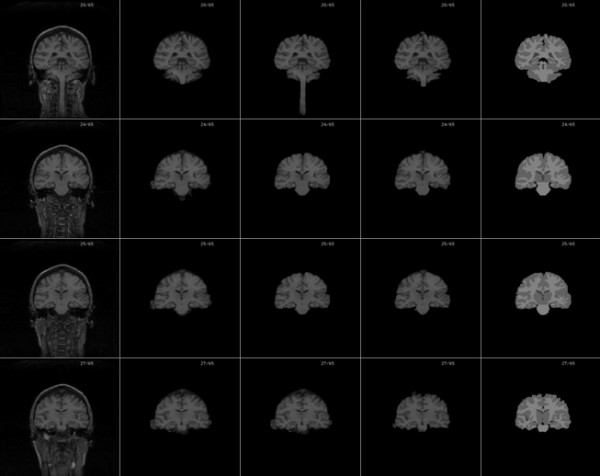
**Comparison results of BET, MLS and the proposed method using four slices of normal T1-weighted MR brain images shown in coronal orientation**. Columns from left to right: raw image, brain extraction results of BET, MLS, our method and manual extraction result.

In addition, we compared our algorithm to two popular brain extraction methods: BET and MLS, using the 10 normal data sets from the IBSR, and the segment results are illustrated in Figure 5. The programs of the BET and MLS were downloaded from their respective WebPages. BET was run on Ubuntu 9.1(a popular Linux distribution). We first ran BET with its default parameters: Fractional Intensity Threshold (FIT, default 0.50) and Vertical Gradient (VG, default 0.0)to segment one training volumes. Unfortunately, with such parameters, BET did not work well, always leading to lots of non-brain tissues(eye, optic nerve, neck.....) being included. Therefore, we did not use the default parameters and by training, the parameters that could provide the best extraction results were applied to all 10 brain volumes (FIT = 0.65, TG = -0.15, robust brain centre estimation). MLS was implemented on the Windows platform using the Java programming language and our algorithm was implemented on the Windows platform using Matlab. The three extraction algorithms were performed on the same hardware platform. Figure 4 shows the comparison results of BET, MLS and the proposed method as well as manual segmentation displayed in coronal orientation. Table 1 lists the comparison result of BET, MLS and the proposed method for multiple indices using the same IBSR data sets. Generally, the larger the sensitivity coefficient, the more accurate the segmentation results. But for a special case, if the segmentation is always conservative, which rather includes lots of non-brain tissues than avoids removing any brain tissue, then FN equals 0 and the sensitivity always equals 1. So an algorithm with larger sensitivity not always have more accurate result. An accurate and robust algorithm must have good performance for multiple indices. Table 1 shows that each algorithm has its advantages and disadvantages. First, the proposed method is superior to BET and MLS with respect to *FP_Rate *and *Specificity*. Second, in our experiments, BET is always conservative and often includes some non-brain tissues, leading to the best sensitivity but worst specificity and *FP_Rate *coefficients. The reason BET had such performance, perhaps because it was more important to avoid removing brain tissue than to remove all non-brain tissues for clinical application. Third, MLS is superior to the our method with regard to sensitivity and has similar performance on Jaccard and Dice indices. Generally speaking, when compared to BET, our method does not include too many non-brain tissues and need not tune many input parameter, so it is accurate and simple to use. Compared to MLS, due to our automatic initialization method, our method is more efficient. So as an automated and simple brain extraction tool, our method can accurately extract brain tissue with high efficiency.

## Conclusions

We proposed an automated and simple brain extraction method using an improved geometric active contour model. Our method has the following advantages over existing brain extraction algorithms: first, Our method uses a binary level set function to eliminate the expensive re-initialization of the existing brain extraction algorithms, it is thus more efficient. Second, the method not only utilizes the image statistical information to construct a new local region-based SPF function, but also corrects the leakage through extremely weak boundaries based on local thresholds estimation, thus can successfully segment brain tissue with weak boundaries. Third, the initial contour can be automatically set inside the brain with sufficiently large radius to improve the automation and the efficiency of the brain extraction. Last but most importantly, our method is very simple and easy to use. No preprocessing step is needed and all the results can be obtained using the original, noisy MR data. Thus, the proposed method can extract brain tissue with high efficiency and full automation compare to two other methods. However, our method was tested using normal adult MRI brain data sets only, and larger sample data sets including different age groups and abnormal anatomy structures such as tumor are needed in order to further test our method as a fully automated, simple and robust method for brain extraction.

## Competing interests

The authors declare that they have no competing interests.

## Authors' contributions

HZ worked on the algorithm design and development, and wrote the paper; JL worked on the algorithm evaluation, ZZ worked on the material preparation and the HL contributed discussions and suggestions throughout this project, including the proofreading the manuscript. All authors read and approved the final manuscript.
